# The relationships between wisdom, positive orientation and health-related behavior in older adults

**DOI:** 10.1038/s41598-023-43868-3

**Published:** 2023-10-04

**Authors:** Magdalena Zadworna, Agnieszka Stetkiewicz-Lewandowicz

**Affiliations:** 1 Institute of Psychology, Faculty of Educational Sciences, University of Lodz, al. Rodziny Scheiblerów 2, 90-128 Lodz, Poland; 2 Department of Medical Psychology, Medical University of Lodz, 90-419 Lodz, Poland

**Keywords:** Psychology, Health care

## Abstract

The concept of healthy ageing, i.e. maintaining health in late life, is closely connected with the role of health behavior. Although health behavior is determined by personal factors, little is known about its relationships with wisdom and positive orientation. Therefore, the aim of the study was to establish relationships between sociodemographic and health factors, personal resources (wisdom and positive orientation) and health-related behavior in late life. The study included 353 Polish seniors aged 60–99 (*M* = 71.95; *SD* = 1.45). The respondents completed the Health-Related Questionnaire for Seniors, Three Dimensional Wisdom Scale, Positive Orientation Scale and a sociodemographic survey. Wisdom and positive orientation were associated with general health behavior and all of its factors. Among the sociodemographic variables, attendance in nonformal education courses had the strongest predictive role for health behavior. A hierarchical regression model demonstrated that personal resources significantly determined healthy lifestyle, after controlling for sociodemographic factors. Additionally, mediational analyses revealed that positive orientation acted as a partial mediator between wisdom and health behavior. Our findings extend knowledge about the factors enhancing healthy lifestyle in older adults, indicating that both wisdom and positive orientation may represent valuable personal resources for health-related behavior.

## Introduction

Recent years have seen increasing attention being paid to the search for factors ensuring identifying people who are *aging well*. Following the first World Report on Aging and Health prepared by WHO^1^, the construct of *healthy aging* is becoming currently widespread used. The definition of healthy aging by the WHO refers to “more than just the absence of disease; it is the process of developing and maintaining the functional ability that enables well-being in older age”*.* Functional ability comprises a number of health-related attributes, viz*.* the intrinsic capacity of the individual, the characteristics of the environment and the interactions between them. In this sense, *intrinsic capacity* refers to genotype, sociodemographic and personal traits, and health characteristics, including health-related behavior, physiological changes, risk factors and diseases.

Health behavior is understood as any activity undertaken by individuals for the purpose of maintaining, enhancing, or protecting their health^2,3^. Health behavior typically includes physical activity, dietary habits, substance avoidance, positive health practices, and preventive behavior. These modifiable behavioral factors appear to have a direct association with the maintenance of objective and subjective health outcomes and life expectancy^4–7^. However, in the late-life period, health behavior may change in response to deteriorating health, loss of physical strength and specific developmental tasks. Older adults demonstrate different health behaviors to other life stages, being more extensively focused on medical treatment, attitudes to intellectual and mental health^8^.

According to theoretical approaches to health behavior determinants, personal traits, self-control abilities, knowledge and beliefs can help foster healthier behavioral adjustments^2,9^. These include sociodemographic variables such as age, sex, former and current education, marital, financial and health status; all of which can affect the level of health behavior^10,11^. A particularly important group of health behavior predictors comprises personal resources. Research has found typical health behavior in older adults to be determined by personal attributes, such as resiliency, spirituality, optimism, and developmental task attainment^12–14^. In later life, personal traits may also affect health behavior by enhancing well-being^12,13^.

One personal resource known to enhance successful adaptation to old age, is wisdom. Among the most widespread psychological theories see^15^, wisdom can be operationalized as a constellation of personality attributes. The current study adopts the three-dimensional approach to wisdom proposed by Ardelt^16^, in which wisdom is a personality trait consisting of three dimensions: cognitive, reflective, and compassionate. The cognitive component makes it easier to fully comprehend commonplace circumstances and to be able to deal with ambiguity when making important judgments. The reflective dimension, on which the cognitive and compassionate components are built, is characterized by the ability to perceive things from a variety of angles. It also calls for self-reflection, self-awareness, and self-insight. The compassionate component refers to having empathy and sympathy for other people.

Wisdom is traditionally associated with health and positive aging^17^. Webster et al.^18^ suggest that wisdom can be associated with health through its influence on health-promoting behavior. Wisdom was found to play a crucial role in choosing adaptive coping strategies, decisions and prosocial behavior, thus strengthening the ability of seniors to cope with aging-related losses^16^. According to selection, optimization, and compensation theory (SOC) proposed by Baltes and Baltes^19^, successful development depends on the processes of investing and managing one's own resources. In old age, when individuals face diminishing resources and changing personal needs, goal selection ensures that available resources are suitably matched to individual preferences for fostering positive functioning. When resources demonstrate a disproportionate loss in comparison to gains, it is important to acquire new resources, or activate previously unused ones, to optimize activities in selected spheres of life and thus compensate for such limitations and losses^20^. The SOC model allows life management to be described and predicted across adulthood^21^. Self-regulatory processes based on selective optimization with compensation are related with a healthy lifestyle and health self-regulation^22^.

From the point of view of the SOC model, wisdom may act as an important resource facilitating various selection, optimization, and compensation processes in later life. Wisdom enhance the ability to take control of one’s environment by purposefully selecting different options or goals in order to achieve a desired end^23^. Thus, wisdom may play a significant role in fostering a healthy lifestyle by selecting the appropriate pro-health decisions and activities tailored to age. Additionally, individuals characterized by greater wisdom tend to approach life’s uncertainties with equanimity^24^. This approach can reduce stress, enhance subjective well-being, and promote physical health. Older adults demonstrating wisdom often exhibit improved self-care, avoid health-adversing habits like smoking and excessive drinking, and engage in health-promoting activities such as physical activity, mindfulness practices, and a balanced diet. However, there is a lack of research on the role of wisdom in promoting health-related behavior.

Another important personal resource is a positive orientation. A positive orientation reflects a basic disposition to appraise life and experiences with a positive outlook; it helps people to cope with life despite adversities, failures and loss. It combines three components: optimism, self-esteem, and satisfaction with life^25^. Such a view is helpful for all people, allowing them to flourish despite the decline of age and the prospect of death^26^. A positive orientation can be considered to be a “good functioning syndrome”, a basis for hedonistic and eudemonistic senses of happiness, and one that correlates positively with the general state of health and well-being across various domains of functioning^27,28^. It has been established that positivity plays an adaptive role in various life domains including social relationships, health issues or coping strategies^29^. The associations between positive mental states and good health may also be bidirectional and partially explained by health-promoting behavior^30^. Positivity has been found to potentially influence health behavior in groups of chronically ill patients^31^, older patients with atherosclerosis^32^ and nurses^33^. However, while few studies have connected a positive orientation with health behavior, many have examined the effects of its components, e.g. optimism^14,34^, self-esteem^35,36^ and satisfaction with life^11,13^. Being also strongly related with self-efficacy, a positive orientation also stimulates positive affect and enhances engagement in life pursuits, including maintaining health^37,38^. By maintaining positive affectivity, an optimistic perspective for the future, engagement, goal setting and self-efficacy beliefs, a positive orientation may also support optimal functioning and serve as a protective factor when people are faced with difficulties and demanding circumstances^28^. Those pathways may explain why people with high levels of positive orientation tend to undertake health-promoting behaviors.

To determine the mechanisms behind the impact of personal resources on health behavior and health outcomes, a good resource is Fredrikson’s^39^ broaden-and-build theory of positive emotions. The theory highlights the significance of positive emotions for physical and psychological well-being. As wisdom and positive orientation are associated with the occurrence of positive emotions, they can strengthen health and well-being. Therefore, better health outcomes may be achieved by health-promoting behavior.

Research has found wisdom to be positively related with well-being, happiness, life satisfaction and resilience^16,40,41^. This influence also occurs in the late life period. Wisdom provides a variety of psychosocial strengths that allow seniors to respond optimally to stressful situations and adapt to developmental tasks. Wiser individuals are more likely to cultivate, nurture and exercise positive social exchanges, which has benefits for both themselves and others. Such people also tend to maintain feelings of self-efficacy, meaning, and purpose in life^18,42^, resilience, mastery and. equanimity^43^. Therefore, wisdom seems to strengthen aspects of “good functioning” similar to the components of positive orientation. Wisdom may help older adults to maintain a sense of emotional well-being despite aging-related losses, i.e. by strengthening other personal resources, stimulating positive and reducing negative affects^44^. Research also suggests that positive orientation can be strengthened in various aspects of life through the development of self-efficacy beliefs, affect regulation and social interactions, and that these can help maintain and enhance optimism, life satisfaction, and self-esteem^28,45^. However, no research has yet focused on the relationships between wisdom and positive orientation construct, nor between wisdom and health-related behavior in later life The current study aims to fill this gap.

Moreover, the potential relationships between wisdom, positive orientation and healthy lifestyle, raise the question of the mediating role of positive orientation in the relationship between wisdom and health-related behavior. Indeed, satisfaction with life, one of the components of positive orientation, was found to mediate the relationship between personal resources and health behavior in older adults^12,13^. Increased life satisfaction may motivate individuals to pursue positive life results and persevere in achieving their goals, potentially leading to a healthier lifestyle.

Therefore, the aim of the current study is to assess the impact of two personal resources, viz*.* wisdom and positive orientation, as well as sociodemographic and health factors, on healthy lifestyle among Polish seniors. The second aim was to examine whether positive orientation mediates the relationship between wisdom and health-related behavior.

We hypothesize that:

1. Wisdom will be correlated positively with health behavior.
2. Positive orientation will be correlated positively with health behavior.
3. After controlling for demographic and health variables, personal resources will improve hierarchical regression models that predict variance in health behavior.
4. Positive orientation will act as a mediator between wisdom and health behavior.

## Methods

### Procedure and data collection

The study was authorized by the Ethics Committee of the University of Lodz. The inclusion criteria were as follows: age over 60 years old and informed consent to participate in the study. The exclusion criteria comprised age below 60, lack of informed consent or incompletely filled questionnaires with several missing responses. The study has a cross-sectional design with convenience sampling. The research was conducted by trained research team members in senior-focused institutions, i.e. community groups like senior clubs, sports clubs, religious societies, healthcare outpatient centers and the University of the Third Age, as well as in participants’ homes; recruitment was also performed via snowball sampling, to ensure diversity in the older adult population. Half of the participants (50.6%) were surveyed in a group setting, attending classes at the University of the Third Age, while the remaining participants completed the survey individually. Informed consent was obtained by all participants. Missing data was accommodated using listwise deletion. From 398 older adults who were approached, 45 did not meet the qualification criteria. The final analysis included results from 353 seniors recruited from central Poland, primarily the Lodz agglomeration and voivodeship, including a large city, small towns and villages. The population of the voivodeship is composed of 28.3% of people aged 60 + , which is close to the mean percentage of seniors in the general population of Poland (25.7%)^46^.

### Participants

The study included 353 Polish seniors aged 60–99 (*M* = 71.95; *SD* = 1.45). Most participants were woman (n = 262, 74.5%). Regarding educational background, 9.3% of participants declared completing primary education, 16.4% vocational school, 41.4% high school, and 32.9% higher education. The majority of the participants were married (45.9%), 90.1% were retired from 1 to 40 years (with an average of 11.56 years). The majority assessed their economic satisfaction as average (56.1%) or good (36%). In addition, 65.4% declared having a chronic illness, and 56.8% reported current attendance in lifelong-leaning education courses and activities, with the most common being the University of Third Age.

The demographic structure of the sample was similar to the general population of seniors in Poland^46^ attending the most typical form of lifelong learning in Poland, i.e. the University of the Third Age^47,48^, with a slight overrepresentation of women.

### Study tools

*Health behavior* was measured using the Health-Related Behavior Questionnaire for Seniors, in a Polish-language version^8^. This 24-item tool measures the health behaviors of older people aged 60 and above according to one overall score for health behavior, together with separate scores for five behavioral categories: (1) Positive attitude towards life – behavior strengthening mental health, such as calmly expressing emotions, or thinking positively about life; (2) Behavior associated with physical health—related to the somatic aspect of health, such as efforts to maintain a normal body weight, avoiding sedentary behaviors or passive smoking; (3) Attention to mental health—behavior aimed at improving intellectual functioning, such as broadening knowledge, or acquiring new skills; (4) Behavior related to prevention and treatment—treatment-related behaviors and illness avoidance, such as following periodic medical examinations, reporting symptoms to a doctor and health information retrieval, and (5) Environmental behaviour—behavior relating to the domain of public health, including environmental protection, such as conserving water and electricity, segregating rubbish and considering the impact of the products on the environment. Answers are given on a 5-point Likert scale. The Cronbach’s alpha coefficient in the current study for the general result was 0.920, for factors 0.770, 0.808, 0.806, 0.782, 0.684, respectively.

*Wisdom* was measured by the Three-Dimensional Wisdom Scale (3D-WS) by Ardelt^16^ in the Polish version of the tool^49^. The scale consists of 39 items, yielding a general score and three dimensions: cognitive (capacity for a comprehensive understanding of life), compassionate (ability for sympathy, compassion to others, and efforts to overcome egocentric tendencies) and reflective (capacity to perceive reality objectively, and evaluate events from various angles). Answers are given on a 5-point scale. The average of the three dimensions and a composite three-dimensional wisdom score were calculated. In the current study, the internal consistency of the tool was satisfactory (Cronbach’s alpha was 0.869 for the general score, and 0.749, 0.710 and 0.672 for the three respective dimensions).

*Positive orientation* was measured by the P-Scale^25^ in the Polish version^37^. The overall result of this eight-item tool is considered as a higher-order latent variable that reflects self-esteem, life satisfaction and optimism. Participants rate their positivity level using a 5-point scale. The tool demonstrated good psychometric properties and excellent internal consistency in the current study (Cronbach’s alpha was 0.848).

*Sociodemographic and health status* was assessed by a questionnaire developed for the purpose of the current study, which collected information about sex (0 = *male*, 1 = *female*), age (*number of years*), education level (1 = *primary*, 2 = *basic vocational*, 3 = *secondary*, 4 = *higher*), marital status (0 = *single*, 1 = *married*), subjectively-perceived financial status (1 = *very bad*, 2 = *bad*, 3 = *average*, 4 = *good*, 5 = *very good*), current professional activity (0-*no*, 1-*yes*) and nonformal education attainment (0 = *no*, 1 = *yes*), and current chronic illness (0-*no*, 1 = *yes*).

### Statistical analysis

The results were analyzed with SPSS Version 25.0. First, demographic characteristics were established using descriptive statistics. The assumption of normality was checked using kurtosis and skewness scores, with their cut points for the normality. Skewness <|2| and kurtosis <|7| indicate a normal distribution.

Then, Pearson’s correlations between variables were also established. Next, a linear hierarchical regression model was used to check for the occurrence of multicollinearity, identify outliers, and adjust for potential confounders. The hierarchical design was intended to contrast personal resources after controlling for sociodemographic and health variables.

Multicollinearity was detected using Variance Inflation Factor (VIF) and tolerance values; following the general rule, it was assumed that the VIF values should not exceed 5.0 and tolerance values should not be less than 0.1 The presence of outliers was detected based on a Mahalanobis distance with the criterion of p < 0.001 and Cook’s distance with case values lower than 1^50^.

Finally, the mediation analysis was performed using PROCESS macro version 4.2. for SPSS, based on a bootstrapping method with 5000 bootstrap samples^51^.

The minimum sample size was determined using G*Power 3.1. software^52^. The level of statistical significance was assumed to be p < 0.05.

### Ethical approval

The study was conducted according to the guidelines of The Declaration of Helsinki, and approved by the Committee for Ethics of Scientific Research at the University of Lodz (1/KBBN-UŁ/I/2019).

## Results

Descriptive statistics were calculated for the variables in the study, as well as a correlation matrix. The analysis included the scores of the psychometric tools (see Table 1).

**Table 1:** Descriptive statistics and correlation matrix for the variables in the study.

Variables	*M*	*SD*	1	2	3	4	5	6	7	8	9	10	11
1. Positive orientation	125.81	18.48	–										
2. Wisdom – general result	3.15	.46	.35 **	–									
3. Cognitive wisdom	3.00	.57	.12 *	.85 **	–								
4. Compassionate wisdom	3.13	.54	.35 **	.87 **	.57 **	–							
5. Reflective wisdom	3.32	.52	.44 **	.83 **	.55 **	.63 **	–						
6. Health-related behavior—overall	94.59	15.33	.52 **	.50 **	.30 **	.46 **	.53 **	–					
7. Positive attitude towards life	23.78	4.20	.60 **	.49 **	.26 **	.48 **	.54 **	.84 **	–				
8. Behavior associated with physical health	23.07	4.93	.39 **	.45 **	.28 **	.41 **	.46 **	.85 **	.60 **	–			
9. Attention to mental health	16.26	3.47	.37 **	.49 **	.38 **	.42 **	.47 **	.80 **	.60 **	.60 **	–		
10. Behavior related to prevention and treatment	16.33	3.32	.35 **	.25 **	.11 *	.26 **	.28 **	.75 **	.55 **	.58 **	.48 **	–	
11. Environmental behavior	15.14	3.23	.31 **	.28 **	.16**	.24 **	.32 **	.73 **	.56 **	.51 **	.53 **	.39 **	–

*M*—mean, *SD*—standard deviation, *p < .05; **p < .01.

Since most factors for skewness and kurtosis were found to be < 2, all were assumed to be normally distributed. The outcomes of the correlation analyses showed significant relationships between all factors, mostly at the level of *p* < 0.001. More specifically, wisdom was correlated with health behavior, and the highest coefficients were obtained for general wisdom and the *attention to mental health* factor of health behavior (*r* = 0.49; *p* < 0.001) and for general health behavior and the reflective dimension of wisdom (*r* = 0.53; *p* < 0.001). Positive orientation was also found to be correlated with health behavior, both the general result and all component factors, with the strongest relationship observed with a *positive attitude toward life* (*r* = 0.60; *p* < 0.001). Positive orientation also correlated positively with general wisdom and all wisdom dimensions. The highest correlation value was obtained between positive orientation and the reflective dimension of wisdom (*r* = 0.44; *p* < 0.001).

Multiple linear hierarchical regression was conducted to find factors predictive for general health behavior (see Table 2). The regression outcomes showed no multicollinearity, indicated by a variance inflation factor (VIF) of 1.03 to 1.42, and tolerance from 0.71 to 0.97. The Durbin-Watson score was 2.04, indicating no autocorrelation in the residuals. The Mahalanobis and Cook’s distance indicated the absence of outliers.

**Table 2:** Summary of the hierarchical regression analysis for variables predicting general health behavior.

Variable	Model 1			Model 2			Model 3		
	*B*	*SE B*	*β*	*B*	*SE B*	*β*	*B*	*SE B*	*β*
Sociodemogaphic and health factors									
Sex^a^	7.35	1.67	.21**	6.11	1.57	.17**	-6.98	1.40	.20**
Age^b^	.06	.11	.03	.06	.10	.03	.08	.09	.04
Education^c^	1.74	.85	.11*	1.08	.79	.07	1.75	.71	.11*
Marital status^d^	3.09	1.49	.10*	2.00	1.40	.06	2.48	1.25	.08
Financial status^e^	2.50	1.20	.11*	1.99	1.12	.08	1.74	1.00	.07
Proffesionally active^f^	1.54	2.45	.03	2.30	2.28	.04	1.65	2.04	.03
Current nonformal education^g^	11.10	1.53	.36**	7.22	1.53	.23**	5.95	1.37	.19**
Chronic ilness^h^	.86	1.59	.03	1.26	1.49	.04	-.64	1.34	-.02
Personal resources									
Wisdom				.29	.04	.35**	.18	.04	.21**
Positive orientation							1.09	.12	.39**
*R*^2^	.26			.36			.49		
*R*^2^ change	.26			.10			.13		
*F* for change in *R*^2^	15.22**			51.84**			86.27**		

^a^Male/female; ^b^number of years; ^c^primary/basic vocational/secondary/higher; ^d^single/married; ^e^very bad/bad/average//good/very good; ^fgh^no/yes; *B*—non-standardized regression coefficients; *SE B*—non-standardized regression coefficients error; *β*—standardized regression coefficient; *R*^2^—determination coefficient; *F*-value of *F*-statistic; **p < .01; *p < .05.

In Model 1, the demographic and health factors predicted 26% of general health behavior variability, with the most important being current attending nonformal education courses, sex (female), marital, financial status and former education status: higher education, higher financial status and being married was associated with better health behavior. Adding the new variable in Model 2, i.e. wisdom, resulted in significant improvement of the model (*β* = 0.35, *t* = 7.20, *p* < 0.001, *R*^2^ change = 0.10). Adding positive orientation in Model 3 improved it also significantly (*β* = 0.39, *t* = 9.29, *p* < 0.001, *R*^2^ change = 0.13), indicating that wisdom and positive orientation has a predictive role for healthy lifestyle, after controlling for sociodemographics and health factors. However, adding positive orientation decreased the predictive role of wisdom in the model. Female sex and current and former education attainment remained significant positive predictors of the dependent variable in the last model. The final model predicted 49% of general health behavior variability.

Finally, the mediation analysis was performed, after controlling for all sociodemographic and health variables, which were significant predictors of the mediator and outcome variable (i.e., sex, attendance in current nonformal education, education level, chronic illness). The analysis revealed that overall wisdom was significantly related to a higher level of general health behavior (total effect: *βc* = 0.37, *SE* = 1.63, *t* = 7.56, *p* < 0.001) and higher positive orientation (indirect effect: *β*a = 0.35, *SE* = 0.68, *t* = 6.37, *p* < 0.001). Positive orientation was also significantly related to a higher level of health-promoting behavior (indirect effect: *βb* = 0.39, *SE* = 0.12, *t* = 9.24, *p* < 0.001). After the introduction of positive orientation as a mediator, the strength of the relationship between wisdom and health behavior decreased by 37.9% but remained significant (direct effect: *βc'* = 0.23 *SE* = 1.54, *t* = 4.98, *p* < 0.001). This result indicates partial mediation (*R*^2^ = 0.48, *F* = 52.60, *p* < 0.001), confirmed by the Sobel test (*Z* = 5.17 *p* < 0.001). The results indicate that older adults characterized by high wisdom were more likely to present positive orientation, which in turn led to higher levels of health-promoting behavior.

## Discussion

The aim of the current study was to assess the associations of personal resources, viz*.* wisdom and positive orientation, as well as sociodemographic and health factors, with a healthy lifestyle among Polish seniors.

As we hypothesized, both general wisdom and all of its dimensions were correlated with general health behavior and all of its factors. Notably, our results highlight that reflective wisdom plays a critical role in terms of its association with general health behavior and with all factors of health behavior, particularly *positive attitude towards life, behavior associated with physical health* and *attention to mental health.* Wiser older adults tend to undertake more health-promoting behavior, especially activities strengthening mental health, as well as those related to the somatic aspect of health and aimed at improving intellectual functioning. The reflective dimension indicates the individual’s ability to perceive life as it actually is, thus better enabling pro-health choices and activities. A clear perception of reality without any major distortions is mandatory for achieving a deeper understanding of life. Wiser individuals tend to engage in reflective thinking by looking at phenomena and events from different perspectives^16^.

Although little research exists concerning the relationship between wisdom and health-promoting behaviors, some studies have covered similar aspects that seem to be part of the construct of wisdom. Character strengths, wisdom being one of them, were found to predispose the individual towards a more health-oriented lifestyle, increasing the motivation to active living^53^. Developing personal strengths enabled greater levels of health-related activity. Other studies have also indicated relationships between wisdom and the lifestyle patterns of middle-aged women^54^ and abstinent behaviors in women recovering from substance abuse^55^.

In the present study, a positive orientation was also correlated with all factors of health behavior and the general result. As expected, a positive orientation had the greatest influence on behaviors strengthening mental health, such as calmly expressing emotions, or avoiding stressful situations. Nevertheless, a positive orientation was also associated with other spheres of lifestyle, such as behavior associated with physical and intellectual health, prevention and treatment and environmental behavior, albeit to a lesser extent.

The results are consistent with other studies indicating that personal resources constituting a positive orientation, including optimism, self-esteem and life satisfaction, are related to health-promoting behavior. Optimistic people, with high self-esteem and who are satisfied with life, may be more likely to engage in a healthy lifestyle^5,13,14,35,36,56–58^.

Older optimists were more likely to be non-smokers, moderate alcohol drinkers, and more likely to engage in exercise. Optimism may promote healthy aging while pessimism may be indirectly conducive for diseases and increase the risk of death by favouring unhealthy behavior^59,60^. Much less is known about the role of positive orientation as a complex construct for health behaviors. Only a few studies have examined the relationship between positive orientation and health behavior^31,33^. Significant associations were noted between positive orientation and health-related behavior in Polish seniors diagnosed with atherosclerosis^32^. Patients with a more positive orientation were more likely to undertake health-favorable behaviors. This result underlines the importance of positive orientation in the treatment process. Substantial scientific evidence links positive emotional health to lower morbidity and mortality; it is possible that in individuals with higher levels of positive emotional resources, these relationships are supported by enhanced health behavior, direct physiological effects and enhanced resistance to stress^61^. The role of positive emotions in health maintenance is also emphasized in broaden-and build theory^39^.

Our findings also confirm our hypothesis that after controlling for demographic and health variables, adding personal resources improves the percentage of variance explained in health behavior. Although the introduction of personal recourses to the regression model improved it significantly, some sociodemographic and lifelong learning factors remained significant predictors: female sex, education level and most importantly, current attendance at nonformal education courses, the most common example being the University of the Third Age. These results are consistent with previous studies indicating that the University of the Third Age attendees presented significantly higher scores for health behavior than their peers not affiliated to any educational organization^11^. Lifelong learning can be an essential element of adaptation to old age, enhancing health-promoting activities through social participation, developing interests, classroom-based informal social support networks and raised awareness. Other studies have also confirmed that various sociodemographic factors, viz*.* female sex, higher level of education and satisfaction with economic status, play a role in taking care of own health in seniors^11^. Other research findings in Polish older adults also showed, that health behavior has multifactor determinants, and personal resources are notably important among them^12,14^.

Finally, mediation analyses revealed that a positive orientation acted as a partial mediator between general wisdom and the overall result of health behavior, after controlling for sociodemographic and health covariates. This seems consistent with a large body of evidence indicating that wisdom is associated with satisfaction with life, a component of positive orientation^16,17,62^. Wisdom encompasses better general physical and mental health, lower risk of alienation, loneliness and depression, and improved health choices, coping strategies and decision-making^17,40,63^. A longitudinal study by Ardelt^17^ revealed that wise individuals age more successfully than people low on wisdom; such people were not only more satisfied during their later years of life, but they also tended to be healthier and to have better family relationships than those with a lower wisdom score. Furthermore, wisdom had a higher impact on life satisfaction than social relations or objective life conditions; a crucial role in this process may be played by emotional regulation, as a component of wisdom. This implies that wisdom may enhance positive orientation resources in older adults, which in turn supports the maintenance of health-promoting behavior. Caprara^28^ suggests that future research should explore the relationships between positivity and constructs that evaluate subjective well-being from a broader perspective, such as mature happiness or mental toughness. Considering the relationship between wisdom and well-being, wisdom appears to be one such construct. Although positive orientation is a relatively stable disposition, Caprara proposes that it may be possible to modify its level. This was confirmed by previous studies indicating that both individual and environmental factors can play predictive and modifying roles^64–67^. Past research has emphasized the significance of fostering competence and self-confidence in managing emotions and interpersonal relationships^68^.

However, research indicates that the relationships formed between well-being, lifestyle and personal resources are of a complex nature. Caprara, Di Giunta, and Caprara^69^ revealed that older people demonstrating greater positivity seem less inclined to report health problems associated with aging. The findings also showed that although positive orientation appears to have a large main effect on healthy aging, the unique impact that its components may still exert should not be overlooked. Life satisfaction showed a significant effect on health over and above the positive orientation. Thus, at older ages, life satisfaction, rather than self-esteem and optimism, appears to be more closely associated with fewer health problems.

Caprara, Di Giunta, and Caprara^69^ also note that wisdom can assist people in aging well. Indeed, being satisfied with one’s life may be viewed as an effect of wisdom encouraging individuals to foster self-esteem and optimism. Grant, Wardle and Steptoe^30^ propose also that the association between satisfaction with life and health-promoting behavior is likely to be bidirectional, but may partly account for the relationship between positive states and good health.

Maintaining healthy behaviors throughout life is believed to reduce the risk of diseases, improve physical and mental capacity and delay care dependency. Therefore, our findings may have important implications for promoting healthy aging. Wisdom is considered to be amenable to intervention^70^ and thus can be trained and cultivated, just like positive orientation^28^. Under this notion, when combined with health education, a tailored intervention targeting the three dimensions of wisdom, self-esteem, optimism and satisfaction with life can support the cultivation of a healthy lifestyle in older adults. The SOC model can serve as a basis for intervention associated with changes in health behavior in old age, thus integrating health- and geropsychological knowledge^71^. Such interventions should also reflect developmental aspects like the changing goals due to decreasing health resources.

A key strength of the current study is that it fills the gap in existing research on personal resources and healthy lifestyle in older adults by exploring the role of wisdom and positive orientation. It also emphasizes the interrelationships between the abovementioned resources, providing new data regarding the partially mediating role played by positive orientation in the relationships between wisdom and health-related behavior. Our findings also highlight the role of lifelong learning in supporting a healthy lifestyle in old age.

Nevertheless, the study has some limitations. Its design is vulnerable to selection bias. It was conducted through convenience sampling in Poland (Central-Eastern Europe) with a predominance of active seniors and attendees of education courses with relatively high socio-economic status, which limits the representativeness of the study. Consequently, it is advisable for future research to focus on recruiting older adults who may be socially isolated and/or have lower levels of socio-economic status.

The cross-sectional character of the study precludes causal conclusions. It also uses quantitative self-reported measures, and health behavior was measured with a Polish tool dedicated strictly to seniors. Hence, the conclusions derived from the current analyses should be regarded with some caution, particularly when applying them to different populations and cultural settings. Moreover, it should be noted that it is possible that the relationships between variables may have a different configuration, as other models with bidirectional pathways between variables are also possible. Further research, including longitudinal studies, is required to identify the factors promoting healthy aging more precisely.

## Conclusions

The current study demonstrates that personal resources are related to health-promoting behavior in older adults, and positive orientation partially mediates the relationship between wisdom and healthy lifestyle. The study makes a useful contribution to the growing literature on the role of wisdom and positive orientation in healthy aging. The findings provide a greater insight into the relationships between personal resources and health-related behavior in the late-life period. They may be of value in both research and clinical practice and offer a new perspective in understanding healthy lifestyle in old age from the psychological point of view.

## Data Availability

The datasets used and/or analyzed during the current study are available from the corresponding author upon reasonable request.
